# Evaluation of Cheek Edema in an Infant Reveals Langerhans Cell Histiocytosis

**DOI:** 10.4314/ejhs.v32i1.24

**Published:** 2022-01

**Authors:** Asimakis D Asimakopoulos, Eugene Panosetti, Alexandra Papoudou-Bai, Chrissa Sioka

**Affiliations:** 1 Department of Oto-Rhino-Laryngology & Head and Neck Surgery, Hospital Centre of Luxembourg, Luxembourg; 2 Department of Pathology, Faculty of Medicine, School of Health Sciences, University of Ioannina, Ioannina, Greece; 3 Department of Nuclear Medicine, University of Ioannina, Ioannina, Greece

**Keywords:** Langerhans cell histiocytosis, osteolysis, PET, CT, imaging

## Abstract

**Background:**

Langerhans cell histiocytosis is a rare hematological disorder. Skin rash is the typical early feature, and bony involvement is the second most common presentation.

**Methods:**

We present a case of a 5-month-old female infant with left hemifacial swelling, initially treated for infection with antibiotics. However, due to persistence of swelling and new onset fever, further evaluation with ultrasonography, CT scan, FDG PET/CT and eventually biopsy was performed.

**Results:**

Imaging methods revealed mandibular osteolysis indicative of either osteomyelitis or histiocytosis X. Tissue biopsy was diagnostic for Langerhans cell histiocytosis.

**Conclusion:**

Langerhans cell histiocytosis may present in infancy with a variety of symptoms, included an isolated bony lesion. Langerhans cell histiocytosis, despite its rarity, should be included in the differentiated diagnosis, when bone osteolysis is found.

## Introduction

Langerhans cell histiocytosis (LCH) is a systemic disease of young age, usually presented with s skin rash but it may involve several organs, such as gastrointestinal tract, liver, spleen, lungs. Tissue biopsy reveals propagation of abnormal Langerhans cells. Prognosis depends on the organs involved (1).

## Case Report

A 5-month-old female infant with left hemifacial swelling for 4 weeks was brought to the outpatient pediatric hospital clinic for evaluation. Initially, even though no specific etiology was evident, the infant was treated with antibiotics for a possible unspecified infection, resulting in a slight decrease of tumefaction. Fifteen days later an urgent consultation was obtained by the Ear Nose and Throat department for persistence of the tumefaction and appearance of fever. Physical examination showed no change of color locally or spontaneous pain, but there was an indurated part in the pre-auricular region and the affected area was sensitive to palpation. Further evaluation with ultrasonography was performed that revealed an hypoechogenic solid mass, slightly vascularized, measured 33x13x17 mm emerging from the left inferior mandible. In addition, there were few hyperechogenic images adjacent to the bone contour which appeared destroyed.

**Figure 1:**
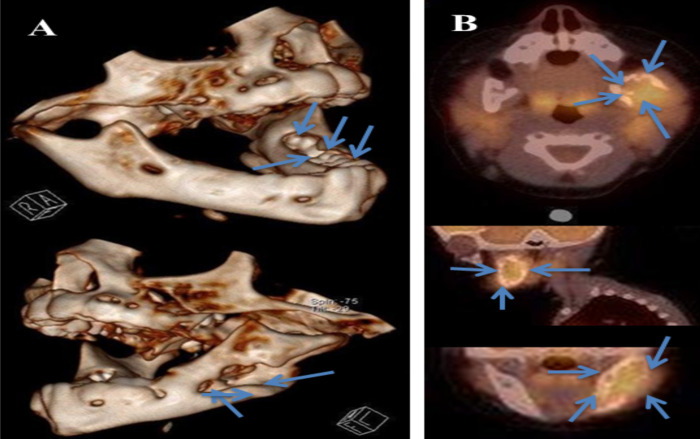
A. CT scan showed peri-apical osteolysis of several teeth on left mandible (arrows). The osteolytic lesion occupying the cortex of left hemimandible, chin spine, the body, ramus and the left mandibular condyle measured 4x2x3cm. This lesion demonstrated low enhancement with contrast media and seemed respecting the adjacent soft tissues. Slight pushback of fatty parapharyngeal space was seen, without important obliteration or densification. There was no mass effect on the level of oropharyngeal pathway or adenopathy. B. FDG PET/CT revealed an osteolytic lesion of left mandible (arrows).

An evaluation with computed tomography (CT) scan was followed, revealing a large osteolytic lesion of the left mandible without categorical invasion of the neighboring soft tissue parts. In absence of an inflammatory clinical entity or of a typical osteomyelitis imaging, histiocytosis X was considered as a probable diagnosis (Figure 1A). Fluorodeoxyglucose-positron emission tomography/computed tomography (FDG-PET/CT) which was followed 3 days later, demonstrated the voluminous osteolytic lesion of the left mandible compatible with either osteomyelitis, histiocytosis X or a potentially tumor (Figure 1B).

**Figure 2:**
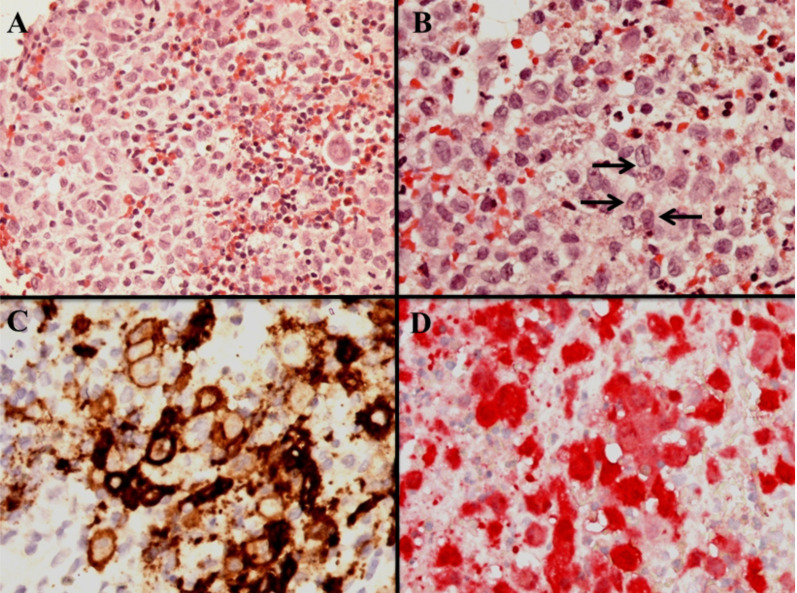
The Langerhans cells were admixed with numerous eosinophils (A, hematoxylin-eosin staining, magnification X400), showing the characteristic nuclear linear grooves (B, black arrows, hematoxylineosin staining, magnification X600). The Langerhans cells expressed CD1a (C, DAB, magnification X600), and S100 (D, alkaline phosphatase, magnification X600). The staining for S100 protein was both cytoplasmic and nuclear.

A transoral biopsy of a recently appeared granuloma-like lesion along the gingival sulcus was performed under general anesthesia. A biopsy of the normal bone-lesional tissue interface revealed Langerhans cell histiocytosis. The biopsy showed numerous Langerhans cells with oval, grooved, folded nuclei and moderately abundant cytoplasm admixed with eosinophils, neutrophils, small lymphocytes and sparse plasma cells (Figure 2).

Since the disease involved only one site in the mandibular area, a combination therapy consisted of prednisone and vinblastine was administered weekly. At present a follow up after 8 weeks confirmed a clear decrease of the volume of left hemimandible mass.

## Discussion

Langerhans cell histiocytosis represents a rare clinical entity with a characteristic pathology. In the neonatal population may range from an isolated bony lesion to a serious systemic disease. Its variety of clinical manifestations could make the diagnosis challenging requiring rigorous investigations for proper management (2).

The mandibles may be affected in about 20%–30% of all cases making the diagnosis challenging (3). Although FDG-PET/CT represents a valuable imaging tool for both Langerhans and non-Langerhans cell histiocytosis, however, a biopsy is needed to confirm the diagnosis (4,5).

Management of Langerhans cell histiocytosis varies depending on the location and the extend of the disease and may include surgery, radiotherapy and corticosteroids with or without chemotherapy. Chemotherapeutic agents include methotrexate, 6-mercaptopurine, hydroxyurea, vinblastine, cytarabine cladribine and others. In our case, since the lesion involved one site and the patient was an infant, a combination of prednisone with vinblastine was selected which had a favorable response. In many cases with single system involvement there is a cure, however, in cases with multisystem involvement recurrences are frequent and the prognosis may be guarded.

A thorough investigation in an infant with a cheek swelling led to the rare diagnosis of Langerhans cell histiocytosis. Thorough investigation, included a biopsy, should be implemented in suspicious cases since the disease is potentially curable.
